# Control of Whitefly (Hemiptera: Aleyrodidae), *Trialeurodes vaporariorum*, with Electron Beam and X-Ray Radiation of Fresh Strawberies for Export

**DOI:** 10.3390/insects11060337

**Published:** 2020-05-31

**Authors:** Sun-Ran Cho, Soeun Shin, Hyeonmo Ahn, Hyun-Na Koo, Yuri Kim, Gil-Hah Kim

**Affiliations:** 1 Department of Plant Medicine, College of Agriculture, Life and Environment Science, Chungbuk National University, Cheongju 28644, Korea; wonfight@naver.com (S.-R.C.); thdmsalsghks@naver.com (S.S.); hyenmo01@naver.com (H.A.); hyunnakoo@hanmail.net (H.-N.K.); 2 EB Tech Co. Ltd., Dajeon 34028, Korea; yrkim@eb-tech.com

**Keywords:** strawberry, exportation, phytosanitory, *Trialeurodes vaporariorum*

## Abstract

Strawberry (*Fragaria ananassa* Duch) is one of the representative fresh agricultural products exported overseas from South Korea. The greenhouse whitefly (Hemiptera: Aleyrodidae), *Trialeurodes vaporariorum*, is an economically important insect pest of commercial strawberries in South Korea. The objective of the present study was to evaluate the effects of electron beam and X-ray on the development and reproduction of *T. vaporariorum*. To determine the radiation dose as a quarantine treatment for strawberry, *T. vaporariorum* were placed at the top, middle, and bottom location in boxes filled with strawberry fruits and irradiated. Eggs were completely inhibited from hatching at 50 Gy, and adult emergence of 3rd nymphs was completely suppressed at 150 Gy in both electron beam and X-ray. Some adults spawning occurred at 100 Gy. However, *F_1_* hatchability was completely suppressed. The results suggest that *T. vaporariorum* was the most radiotolerant to both of ionization energy at the nymph stage. The dosimetry results showed that the penetrating power of ionizing radiation in boxes filled with strawberry fruits was the lowest at the bottom location. A treatment dose of 150 Gy is adaptable as a quarantine treatment to *T. vaporariorum* nymph in strawberry fruit. Our results indicate that ionizing radiation could be recommendable as a phytosanitary treatment for quarantine.

## 1. Introduction

Due to the breeding of domestic strawberry varieties in 2003, strawberry (*Fragaria ananassa* Duch) exports from South Korea have been steadily increasing [1]. As of 2017, 41.6% of exported strawberries were exported to Hong Kong, 27.1% to Singapore and 13.5% to Thailand, of a total of 4,788 tons [2]. Because strawberries for export are transported by ship or airplane, ‘Maehyang (*F. ananassa cv.*)’ strawberries, which have superior storage quality, are the main variety exported; ‘Maehyang’ strawberries are cultivated in Korea and have a sweet and sour taste [3,4].

*Trialeurodes vaporariorum* develops rapidly in greenhouse agroecosystems, and the density of whiteflies increases in a geometrical progression [5]. As such, strawberry-growing greenhouses are good places for whiteflies to live [6]. Therefore, it can be harvested and packaged with the eggs, nymphs, and adults of *T. vaporariorum* on the part of the strawberry stem.

Ionizing radiation, including electron beam, X-ray and gamma-ray, can be used for PI (phytosanitary irradiation) and the SIT (sterile insect technique) can also be used [7,8,9]. Irradiation is used to control insects during quarantine periods after the harvest of agricultural products [10,11]. In addition, at doses of radiation that do not harm the quality of agricultural products, radiation can be used extensively and effectively for pest control [12,13,14].

When strawberry fruits are irradiated, their shelf life is extended, and fruit weight loss and decay are reduced compared to those in untreated fruit [15,16]. Additionally, radiation can be performed at low temperatures and reduces damage to fruit, unlike fumigation [17]. Other studies have suggested that blueberries are generally tolerant of radiation [18,19].

Hallman [20] suggested that to control Aleyrodidae, a radiated gamma-ray dose of 100 Gy should be used. Additionally, other research has effectively inhibited reproduction at gamma-ray doses of 50–70 Gy in *T. vaporariorum* pupae and adults [5,21,22,23]. Van Nieuwenhove et al. [24], in a large-scale study, suggested that a target dose of 108 Gy prevented reproduction in *T. vaporariorum*. In previous studies, we applied gamma rays to strawberry boxes packed for export and proposed an inhibitory dose for two species of whiteflies (*Bemisia tabaci* and *T. vaporariorum*) [25]. In addition, the inhibitory doses of electron beam and X-ray radiation for six insect pest species (*Spodoptera litura, Tetranychus urticae, Frankliniella intonsa, Bemisia tabaci, Liriomyza trifolii, Myzus persicae*) [26,27] in boxes of cut-flower roses and chrysanthemums were developed. Additionally, Koo et al. [28] studied the inhibitory doses of X-ray radiation for two species of thrips (*Frankliniella intonsa* and *F. occidentalis*) in boxes of lilies.

The susceptibility of *T. vaporariorum* to gamma-ray radiation has been studied [5,21,22,23,24,25]. However, the effects of electron beam and X-ray radiation on *T. vaporariorum* in different areas of boxes filled with strawberry fruits have not yet been studied. Therefore, the present study will aid in the development of nonchemical methods for insect control using electron beam and X-ray in the preparation of strawberry fruits for export from South Korea.

## 2. Materials and Methods

### 2.1. Test Insects

The whitefly population was collected from a rose greenhouse in Jincheon (Republic of Korea) in May 2010. The pesticide-susceptible strains of *T. vaporariorum* used in this study were reared, beginning in 2010 in a laboratory at Chungbuk National University (Republic of Korea) and were never exposed to pesticides. The *T. vaporariorum* were reared at 25 ± 1 °C, 50–60% RH and a 16:8 h light/dark photoperiod. Tomato seedlings (*Lycopersicon esculentum*) were used as hosts. The principal reason for this is that it grows better in pots than strawberry seedlings, and is easier to experiment for a long time.

### 2.2. Ionizing Radiation Treatment

The electron beam irradiation was conducted at EB-Tech Co., Ltd. (Daejeon, South Korea), using a high-energy linear accelerator (UEL V10-10S, 10 MeV). The X-ray treatment was also conducted at EB-Tech Co., Ltd. using a high-energy linear accelerator (UEL V10-10S, 7.5 MeV). The target doses were monitored by dosimetry with an alanine pellet dosimeter (ES 200-2106, Bruker BioSpin Co., Billerica, MA, USA). The absorbed irradiation doses were set at 50, 70, 100, 150, and 200 Gy. Twenty female and twenty male whitefly adults were placed in a square acrylic cage (30 × 30 × 45 cm) with tomato seedlings. The adults were allowed to lay eggs for 24 h, after which they were removed. One or two tomato leaves with attached eggs (0–24 h old) were used. After irradiation, the leaves were placed in a glass vial (2.5 cm in diameter by 7 cm in height) and monitored for egg hatching for 10 days in a growth chamber (25 ± 1 °C, 50–60% RH and a 16:8 h light/dark photoperiod). To test the effects of irradiation on nymphs, a tomato leaf with 3rd instars was prepared. After irradiation, the newly emerged adults were counted for 14 days. At 0–24 h after emergence, the adults were placed in a glass vial (2.5 cm in diameter by 7 cm in height). After irradiation, pairs of adults were incubated in square cages (7 × 7 × 10 cm) with tomato seedlings. The number of progency from the irradiated adults was recorded daily, and the *F*_1_ hatching rate was observed. All experiments were performed independently three times.

### 2.3. Small Scale-Up Validation Test

**Figure 1:**
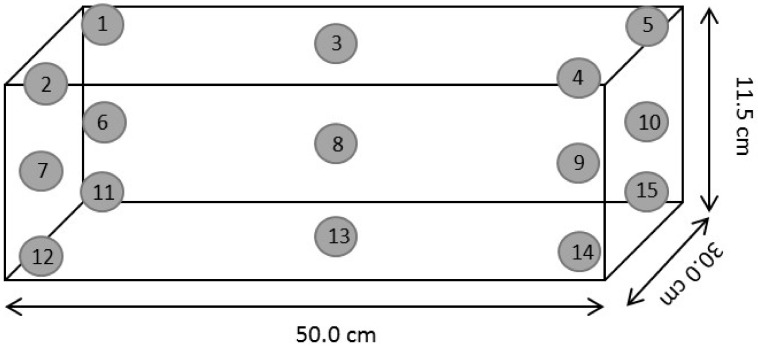
Position of alanine pellets for dose mapping in boxes filled with strawberry fruits.

The ‘Maehyang’ (*F. ananassa cv. Maehyang*) strawberry cultivar was used in the present study and was purchased from the Sogok Dukcheon Agricultural Cooperative Export Group (Jinju, Korea). An alanine pellet (Bruker BioSpin) dosimeter was used to measure the absorbed doses of radiation (electron beam and X-ray). After the boxes were filled with strawberries (total 2 kg = 250 g/pack × 8 ea), the pellets were installed at various positions in the boxes (50 × 30 × 11.5 cm) (Figure 1). The boxes were then packed and irradiated (150, 200 and 300 Gy), and the absorbed doses were measured. After evaluation of the calibration procedures described above, adults (0–24 h after emergence) were placed in a glass vial (2.5 cm in diameter by 7 cm in height), and tomato leaves with eggs (0–24 h old) and nymphs were placed in zippered plastic bags (10 × 16 cm). The vials and bags were placed in the top, middle, bottom part of the strawberry box, according to electron beams and X-rays. After irradiation, bioassays of the *T. vaporariorum* life stages were carried out by the procedure described above. All experiments were performed independently three times.

### 2.4. Data Analysis

Based on the above investigation, the effects of electron beam and X-rays’ irradiation on egg hatch, adult emergence, fecundity, and *F*_1_ egg hatch were compared by one-way analysis of variance (ANOVA) followed by Tukey’s studentized range test. All statistical analyses were conducted using JMP [29].

## 3. Results

### 3.1. Effects of Electron Beam and X-Ray Irradiation on Development Stages

The hatchability, adult emergence rate, fecundity and *F*_1_ hatchability of *T. vaporariorum* treated by ionizing radiation are shown in Table 1 and Table 2. In the untreated control group (0 Gy), the hatching rate of the *T. vaporariorum* eggs was 98.8%. Egg hatching was completely inhibited at 70 Gy in both electron beam and X-ray. The nymphs of *T. vaporariorum* were treated with radiation doses of 50–150 Gy. The emergence rate of adults after the development of 3rd instars decreased with increasing ionizing radiation doses, and emergence was completely inhibited at 150 Gy. When *T. vaporariorum* adults (0–24 h after emergence) were irradiated with 50–100 Gy doses, their fecundity decreased with increasing radiation doses. Additionally, *F_1_* egg hatching failed at 100 Gy and 70 Gy of electron beam and X-ray irradiation, respectively. These results suggest that the most radiotolerant stage in *T. vaporariorum* was the nymph stage.

### 3.2. Dose Mapping at Each Location of Boxes Filled with Strawberry Fruits

The absorbed dose ranges of electron beam and X-ray radiation in boxes filled with strawberry fruits are presented in Table 3 and Table 4. The measured doses were very close to the target doses, except at the bottom location in both electron beam and X-ray (no. 11~15 of Figure 1). At the top, middle, and bottom location of the box, the target doses and the absorbed doses differed in each location of the box. In this study, 300 Gy of electron beam and X-ray irradiation did not change the quality of the strawberry fruits. 

The results of the dose mapping of the electron beam in boxes filled with strawberry fruits showed that the DUR values at 150, 200, and 300 Gy were 2.2, 2.2, and 2.0, respectively (Table 4). The results for the X-rays showed that the DUR values at 150, 200, and 300 Gy were 1.8, 1.6, and 1.7, respectively (Table 5). 

### 3.3. Small Scale-Up Validation Test on T. vaporariorum

The eggs of *T. vaporariorum* were treated with an electron beam and X-ray dose of 200–300 Gy at the top, middle, and bottom location in boxes filled with strawberry fruits (Table 6). Egg hatching by 200 Gy was completely inhibited at all locations. In the untreated control group (0 Gy), the hatching rate of *T. vaporariorum* eggs was 96.3%. 

The nymphs of *T. vaporariorum* were treated with 200–300 Gy doses at the top, middle, and bottom locations in boxes filled with strawberries (Table 7). The emergence rate of untreated *T. vaporariorum* nymphs was 93.6%. The irradiated *T. vaporariorum* nymphs failed to emerge at almost all locations in boxes, except at the bottom at a dose of 200 Gy. However, adult emergence of *T. vaporariorum* was completely inhibited at the bottom of the box, even at a 300 Gy dose. 

When *T. vaporariorum* adults were irradiated with 150–200 Gy of electron beam and X-ray radiation, the fecundity was lower at the bottom of the boxes compared with that in the untreated control group (Table 8). Although the irradiated adults laid eggs, hatching was completely inhibited. In the untreated control group, the hatching rate of *T. vaporariorum* eggs was 92.1%. 

## 4. Discussion

Our results revealed that inhibition of developmental stages of *T. vaporariorum* were increased with doses of electron beam and X-ray radiation. In addition, ionizing radiation could be a phytosanitary treatment for quarantine on strawberry. Similar to the present study, Cho et al. [25] reported that when *T. vaporariorum* and *B. tabaci* were irradiated by gamma-rays, the most radiotolerant stage was the nymph stage. Additionally, males are generally reported to be more radiotolerant than females [23,30,31,32,33,34,35,36].

The absorbed dose varied according to each locations position in the commodity box. This difference occurred because the penetrating power of radiation depends on the density and moisture content of the packaged product [37]. The difference in the absorbed dose in a box filled with products varied depending on the size, number, shape, water content and type of products [25,26,28]). In this study, 300 Gy of electron beam and X-ray irradiation did not change the quality of the strawberries. Previous studies have reported that strawberries may tolerate an irradiation dose up to 2 kGy without changes in quality [15,16,38]. Additionally, Sharma and Rastogi [39] reported the effects of a gamma radiation dose of 1.2 kGy on the quality and shelf life of strawberries. In addition, the gamma-ray radiation on strawberry boxes used for export did not affect strawberries until a dose of 400 Gy [25]. Barkai-Golan and Follett [40] reported that strawberries are very radiotolerant and are an optimal target commodity.

Follet and Lower [41] reported that a change in the target dose can result in false positives for the absorbed dose required for quarantine treatment; therefore, the DUR (dose uniformity ratio, maximum/minimum) must be checked in commercial radiation treatments. Both the electron beam and the X-ray were equal in the value. Our previous results showed that the DUR values in boxes filled with strawberry fruits for gamma ray radiation at 100 to 400 Gy were 1.11 to 1.19 [25]. Follett and Weinert [37] demonstrated that the DUR value in the fruit and in the inner wall of the box was 1.34–1.49, depending on the fruit type. The rate generally ranges from 1.6 to 3.0, but a very high DUR can be difficult to penetrate with the target dose [37,42,43]. Because the dose decreases with distance squared, the greater the depth (width) of the goods or the density of commodities in the box is, the higher the DUR value will be [37,42].

As in our data, when *B. tabaci* eggs were irradiated using 200 Gy of electron beams [26] and 150 Gy of X-rays [27], hatching was completely inhibited at all locations in both the rose and chrysanthemum boxes. Similar to the present study, Yun et al. [27] reported that *B. tabaci* adults irradiated by 150 Gy of X-rays, although some in the three sections (top, middle, bottom) of both the rose and chrysanthemum boxes were able to lay eggs, the *F_1_* generation did not completely hatch. However, the emergence of *B. tabaci* adults was inhibited at every position in both the rose and chrysanthemum boxes at 200 Gy of electron beam irradiation [26]. In addition, Koo et al. [28] suggested that spawning inhibition failed in adults of both *Frankliniella occidentalis* and *F. intonsa* at the bottom positions at X-ray doses of 200 Gy, but the hatchability of the *F_1_* generation was completely suppressed at 300 Gy of X-ray.

An effective quarantine disinfection method does not permit the reproduction of more than 0.01% of individuals [44]. Therefore, we had to find a dose that would completely inhibit the development and reproduction of *T. vaporariorum*. In our small scale-up validation test, a minimum dose of 150 Gy (electron beam and X-ray) is recommended for the control of *T. vaporariorum* in the preparation of strawberry for export.

## 5. Conclusions

A minimum dose of 300 Gy, both for electron beams and X-rays, is recommended for the control of *T. vaporariorum* in the preparation of strawberry boxes for export.

## Figures and Tables

**Table 1:** Effect of electron beam irradiation on the hatchability, emergence, longevity, fecundity, and *F_1_* hatchability of *Trialeurodes vaporariorum.*

Stage	Dose (Gy)	N	Hatchability (%)			Adult Emergence (%)			No. of Eggs per Female			Hatchability (%) (*F*_1_)		
Eggs	150	79	0.0	±	0.0 b ^1^		- ^2^			-			-	
	100	438	0.0	±	0.0 b		-			-			-	
	70	495	0.0	±	0.0 b		-			-			-	
	50	413	0.5	±	0.8 b		-			-			-	
	0	194	98.8	±	2.1 a		-			-			-	
Nymph	150	128		-		0.0	±	0.0 b		-			-	
	100	93		-		2.7	±	2.3 b		-			-	
	70	169		-		22.6	±	33.0 b		-			-	
	50	193		-		35.6	±	40.5 ab		-			-	
	0	88		-		92.6	±	4.9 a		-			-	
Adults	100	60		-			-		6.3	±	11.1 b	0.0	±	0.0 b
	70	60		-			-		13.7	±	11.3 b	1.3	±	3.0 b
	50	60		-			-		15.1	±	9.6 b	2.6	±	6.5 b
	0	60		-			-		34.0	±	15.0 a	94.6	±	6.1 a

All data are Mean ± SD. ^1^ Means within each column followed by the same latter are not significantly different at *p* < 0.05 by Tukey’s studentized range test (SAS Institute, Cary, NC, USA, 2009). ^2^ The dashes mean no data needed or available because of the small number of test insects.

**Table 2:** Effect of x-ray irradiation on the hatchability, emergence, longevity, fecundity, and *F_1_* hatchability of *T. vaporariorum.*

Stage	Dose (Gy)	N	Hatchability (%)			Adult Emergence (%)			No. of Eggs per Female			Hatchability (%) (*F*_1_)		
Eggs	100	613	0.0	±	0.0 b ^1^		- ^2^			-			-	
	70	718	0.0	±	0.0 b		-			-			-	
	50	1480	1.5	±	0.7 b		-			-			-	
	0	194	98.8	±	2.1 a		-			-			-	
Nymph	150	326		-		0.0	±	0.0 c		-			-	
	100	445		-		6.0	±	3.2 c		-			-	
	70	673		-		16.1	±	18.8 c		-			-	
	50	286		-		41.6	±	8.0 b		-			-	
	0	88		-		92.6	±	4.9 a		-			-	
Adults	100	60		-			-		11.3	±	18.1 b	0.0	±	0.0 b
	70	60		-			-		16.4	±	25.1 b	0.0	±	0.0 b
	50	60		-			-		16.7	±	19.3 b	2.9	±	9.1 b
	0	60		-			-		34.0	±	15.0 a	94.6	±	6.1 a

All data are Mean ± SD. ^1^ Means within each column followed by the same latter are not significantly different at *p* < 0.05 by Tukey’s studentized range test (SAS Institute, 2009). ^2^ The dashes mean no data needed or available because of the small number of test insects.

**Table 3:** The absorbed doses of electron beam and X-rays according to their location in boxes filled with strawberry fruits.

Target Dose (Gy)	Position	Absorbed Dose (Gy; Mean ± SD)					
		Electron Beam			X-Ray		
150	Top	161.1	±	11.4 a *	171.1	±	11.5 a
	Middle	129.1	±	9.1 b	140.4	±	5.7 b
	Bottom	90.0	±	6.9 c	122.7	±	10.0 c
200	Top	212.7	±	11.1 a	216.2	±	13.8 a
	Middle	161.3	±	7.1 b	183.5	±	11.4 b
	Bottom	118.9	±	13.0 c	161.9	±	8.9 c
300	Top	312.0	±	10.3 a	326.3	±	10.6 a
	Middle	258.2	±	17.7 b	280.9	±	13.3 b
	Bottom	186.5	±	16.3 c	238.7	±	25.1 c

* Means within each column followed by the same letter are not significantly different at *p* < 0.05 by Tukey’s studentized range test (SAS Institute 2009).

**Table 4:** Dose mapping of electron beam in boxes filled with strawberry fruits.

Position	Pellet No.	Dose (Gy)		
		150	200	300
Top	1	164.7	216.7	298.5
	2	144.9	224.9	326.4
	3	159.2	195.2	308.9
	4	176.5	216.5	316.7
	5	160.4	210.4	309.4
Middle	6	126.5	162.5	241.4
	7	137.6	167.3	280.9
	8	126.4	164.2	244.7
	9	138.4	148.9	273.2
	10	116.5	163.5	251.0
Bottom	11	85.1	115.4	185.5
	12	96.8	116.8	207.9
	13	91.6	135.6	163.0
	14	95.9	100.8	192.7
	15	80.8	125.9	183.5
D_min_		80.8	100.8	163.0
D_max_		176.5	224.9	326.4
Dose uniformity ratio (maximum/minimum)		2.2	2.2	2.0

**Table 5:** Dose mapping of X-ray in boxes filled with strawberry fruits.

Position	Pellet No.	Dose (Gy)		
		150	200	300
Top	1	178.2	212.5	319.8
	2	169.4	206.4	344.3
	3	186.5	237.9	322.8
	4	157.1	203.6	326.7
	5	164.3	220.6	317.8
Middle	6	131.8	193.6	262.5
	7	143.9	171.5	294.5
	8	146.8	197.3	279.4
	9	138.7	174.7	293.0
	10	140.8	180.5	274.9
Bottom	11	128.1	157.4	239.5
	12	125.6	153.0	227.1
	13	122.6	165.9	203.6
	14	105.7	157.9	268.7
	15	131.4	175.4	254.8
D_min_		105.7	153.0	203.6
D_max_		186.5	237.9	344.3
Dose uniformity ratio(maximum/minimum)		1.8	1.6	1.7

**Table 6:** Mean (±SD) egg hatchability (%) of *T. vaporariorum* at three different locations (top, middle, and bottom) in boxes filled with strawberry fruits irradiated by 200, 300 Gy of electron beam and X-rays.

Radiation	Gy	Position	N	Hatchability (%)		
Electron beam	300	Top	371	0.0	±	0.0 b *
		Middle	290	0.0	±	0.0 b
		Bottom	264	0.0	±	0.0 b
	200	Top	475	0.0	±	0.0 b
		Middle	446	0.0	±	0.0 b
		Bottom	449	0.0	±	0.0 b
X-ray	300	Top	362	0.0	±	0.0 b
		Middle	445	0.0	±	0.0 b
		Bottom	391	0.0	±	0.0 b
	200	Top	328	0.0	±	0.0 b
		Middle	349	0.0	±	0.0 b
		Bottom	388	0.0	±	0.0 b
	0		475	96.3	±	2.5 a

* Means within each column followed by the same letter are not significantly different at *p* < 0.05, by Tukey’s studentized range test (SAS Institute 2009).

**Table 7:** Mean (±SD) adult emergence rate from nymph (%) of *T. vaporariorum* at three different locations (top, middle, and bottom) in boxes filled with strawberry fruits irradiated by 200, 300 Gy of electron beam and X-rays.

Radiation	Gy	Position	N	Adult Emergence (%) *		
Electron beam	300	Top	981	0.0	±	0.0 c
		Middle	1102	0.0	±	0.0 c
		Bottom	1120	0.0	±	0.0 c
	200	Top	1085	0.0	±	0.0 c
		Middle	1319	0.0	±	0.0 c
		Bottom	1085	3.7	±	5.3 b
X-ray	300	Top	1324	0.0	±	0.0 c
		Middle	1103	0.0	±	0.0 c
		Bottom	1347	0.0	±	0.0 c
	200	Top	1059	0.0	±	0.0 c
		Middle	1267	0.0	±	0.0 c
		Bottom	1454	2.4	±	1.7 bc
	0		456	93.6	±	1.8 a

* Means within each column followed by the same letter are not significantly different at *P* < 0.05 by Tukey’s studentized range test (SAS Institute 2009).

**Table 8:** Mean (±SD) adult fecundity (no. of eggs per female) hatchability (%) of the eggs (F1 generation) of *T. vaporariorum* irradiated by 150, 200 Gy of electron beam and X-rays at bottom location in boxes filled with strawberry fruits.

Radiation	Gy	Position	N	No. of Eggs per Female			Hatchability (%) (*F*_1_)		
Electron beam	200	Bottom	60	6.2	±	11.0 b *	0.0	±	0.0 b
	150		60	7.1	±	11.3 b	0.0	±	0.0 b
X-ray	200	Bottom	60	5.8	±	13.8 b	0.0	±	0.0 b
	150		60	8.2	±	10.8 b	0.0	±	0.0 b
	0		60	38.0	±	19.6 a	92.1	±	4.9 a

* Means within each column followed by the same letter are not significantly different at *p* < 0.05 by Tukey’s studentized range test (SAS Institute 2009).
